# Accuracy and Completeness of Online Patient Information on Nail-Patella Syndrome: A Pilot Cross-Sectional Study

**DOI:** 10.7759/cureus.115019

**Published:** 2026-08-23

**Authors:** Hazem Mohamed, Dimitrios Manoukian

**Affiliations:** 1 Trauma and Orthopaedics, The Princess Alexandra Hospital National Health Service (NHS) Trust, Harlow, GBR; 2 Trauma and Orthopaedics, The Royal London Hospital, London, GBR

**Keywords:** glaucoma, infodemiology, information accuracy, lmx1b, nail-patella syndrome, nephropathy, online health information, patient education

## Abstract

Background: Nail-patella syndrome is a rare autosomal dominant multisystem disorder with musculoskeletal, renal, and ocular manifestations. Patients and families may rely on online resources, but the completeness and accuracy of this information have not been well characterized. This study evaluated English-language online information about nail-patella syndrome.

Methods: A structured cross-sectional pilot search conducted on August 6, 2026, identified 24 candidate domains. Fifteen independent patient-facing websites were included in the primary analysis. Pages were grouped by source type and assessed using a 15-item Nail-Patella Syndrome Specific Content Score (range 0-15) and an accuracy-completeness score in which each item was rated 0 (absent), 1 (present but incomplete or partly inaccurate), or 2 (present and broadly accurate; range 0-30). GeneReviews and peer-reviewed literature formed the reference standard.

Results: The mean presence score was 13.2 of 15 (standard deviation 1.8; range 10-15), and the mean accuracy-completeness score was 23.7 of 30 (standard deviation 5.3; range 14-30). Government/non-profit websites had the highest mean accuracy-completeness score (26.0), followed by healthcare-provider (25.0), media/general-information (23.7), and commercial websites (20.0); differences were not statistically significant (Kruskal-Wallis H(3)=2.58, p=0.462). Nail, knee, and elbow manifestations were consistently described. Epidemiology was present on eight websites, while diagnosis/investigations, genetic counselling, and surveillance were each present on 11. Renal manifestations were mentioned on all websites, but only 10 were fully accurate; glaucoma was omitted by one website.

Conclusions: This pilot study identified specific gaps in online patient-facing information about nail-patella syndrome, particularly concerning diagnosis, renal and ocular surveillance, and balanced management. These findings highlight areas for improvement in patient resources rather than establishing a definitive ranking of individual websites.

## Introduction

Patients increasingly use the Internet to understand diagnoses and treatment options, yet online medical information varies in authorship, currency, completeness, and clinical accuracy [1-4]. Orthopaedic website evaluations frequently combine general reliability instruments with condition-specific content measures [1,2]. The calcaneal-fracture study by Patel et al. provided a concise model by classifying website sources and applying a disease-specific content score alongside established quality measures [1]. DISCERN and related instruments are useful for treatment-choice information, but their scores depend on judgement and benefit from independent assessment [5,6]. Existing general health-information assessment tools address important aspects of website quality, reliability, readability, or treatment information, but when used alone they do not directly determine whether a resource covers the specific multisystem clinical features of nail-patella syndrome or whether those disease-specific statements are clinically accurate and complete. Because Nail-patella syndrome (NPS) includes musculoskeletal, renal, ocular, genetic, diagnostic, surveillance, and management considerations, a condition-specific content framework was therefore developed to assess whether these clinically important domains were represented and accurately described.

Nail-patella syndrome is a rare autosomal dominant multisystem disorder classically associated with nail dysplasia, abnormal or unstable patellae, elbow abnormalities, and iliac horns [7-10]. Pathogenic variants in the LMX1B gene disrupt limb, renal, and ocular development, although clinical expression varies markedly within and between families [11-14]. Renal involvement may present with proteinuria or haematuria and can progress to chronic kidney disease, while ocular hypertension and glaucoma create a preventable risk to vision [15-19]. Neurological symptoms, reduced skeletal integrity, and substantial knee morbidity have also been described [20-24]. Current management is individualized and multidisciplinary, with long-term renal and ophthalmological surveillance remaining central even when musculoskeletal symptoms dominate the presentation [8,21,25].

The name of the condition may encourage patients to perceive it as a disorder limited to the nails and patellae. Incomplete online information could therefore delay recognition of renal or ocular complications. However, the extent to which publicly available NPS resources accurately and comprehensively address the key clinical domains of this multisystem disorder has not been clearly established. The primary objective of this study was to systematically evaluate the presence, accuracy, and completeness of core nail-patella syndrome information on publicly accessible patient-facing websites and to identify clinical domains commonly omitted or presented incompletely. The secondary objective was to explore descriptive differences in information quality across website source categories.

## Materials and methods

Study design and website selection

This cross-sectional pilot evaluation was reported in accordance with STrengthening the Reporting of OBservational studies in Epidemiology (STROBE) principles [7]. A structured Internet search was conducted from the United Kingdom on August 6, 2026, using Google UK (Google UK Limited, London) through Microsoft Edge (Microsoft, Redmond, WA) on a private laptop running Windows 11. The predefined search phrases were “nail patella syndrome,” “nail patella syndrome symptoms,” “nail patella syndrome diagnosis,” “nail patella syndrome treatment,” “nail patella syndrome kidney glaucoma,” and “nail patella syndrome children.” Searches were conducted using a private browsing session without an active Google account. The first 20 non-sponsored organic results for each query were initially screened; because fewer than 20 eligible independent patient-facing domains were identified, screening was extended uniformly to the first 30 organic results for each query. Results were assessed in the order returned by Google, and where more than one eligible page arose from the same domain, the highest-ranked substantive patient-facing resource was retained. Repeated domains across search queries were consolidated before the final eligibility assessment so that each domain was represented once. Substantially duplicated content across different domains, particularly NHS-derived material, was identified separately and reserved for sensitivity analysis.

Inclusion criteria were: (1) publicly accessible without payment or registration; (2) written in English; (3) directed primarily toward patients, families, or the general public; (4) containing substantive information specifically concerning nail-patella syndrome rather than a brief definition or link directory; and (5) representing an independent information source. Where more than one eligible page was identified from the same domain, only the principal patient-facing resource was assessed.

Exclusion criteria were: (1) research articles or case reports; (2) clinician- or professional-only resources; (3) pages requiring payment or registration; (4) inaccessible or broken webpages; (5) pages redirecting to a resource already represented in the sample; (6) substantially duplicated content, including NHS-derived material, which was excluded from the primary analysis and evaluated separately in sensitivity analysis; and (7) resources used as part of the study reference standard rather than as patient-information websites [8].

Of the 24 candidate domains screened, 15 independent patient-facing websites entered the primary analysis. Of the remaining nine domains, three contained substantially duplicated or NHS-derived material and were reserved for sensitivity analysis, two were clinician-facing resources retained as professional backups, three were inaccessible, broken, or redirected to an already included resource, and GeneReviews was used as the principal reference standard rather than being scored as a patient-facing website.

Website classification and scoring

Websites were categorized as government/non-profit, healthcare provider, commercial, or media/general information. A 15-item Nail-Patella Syndrome Specific Content Score was adapted from the condition-specific approach used by Patel et al. [1]. One point was awarded when a topic was substantively present, producing a presence score from 0 to 15. Each item was also rated 0 when absent, one when present but incomplete, outdated, overgeneralized, or partly inaccurate, and 2 when present and broadly consistent with the reference standard, producing an accuracy-completeness score from 0 to 30. The assessed domains are listed in Table 1.

**Table 1:** Nail-patella syndrome specific content score

Item	Information domain	Scoring rule
1	Definition and multisystem nature	Presence: 0-1; accuracy-completeness: 0-2
2	Etiology and LMX1B gene	Presence: 0-1; accuracy-completeness: 0-2
3	Inheritance	Presence: 0-1; accuracy-completeness: 0-2
4	Epidemiology	Presence: 0-1; accuracy-completeness: 0-2
5	Age and variability of presentation	Presence: 0-1; accuracy-completeness: 0-2
6	Nail manifestations	Presence: 0-1; accuracy-completeness: 0-2
7	Patellar and knee manifestations	Presence: 0-1; accuracy-completeness: 0-2
8	Elbow and radial-head manifestations	Presence: 0-1; accuracy-completeness: 0-2
9	Iliac horns and pelvic manifestations	Presence: 0-1; accuracy-completeness: 0-2
10	Renal manifestations	Presence: 0-1; accuracy-completeness: 0-2
11	Ocular manifestations	Presence: 0-1; accuracy-completeness: 0-2
12	Diagnosis and investigations	Presence: 0-1; accuracy-completeness: 0-2
13	Genetic counselling and family implications	Presence: 0-1; accuracy-completeness: 0-2
14	Surveillance	Presence: 0-1; accuracy-completeness: 0-2
15	Treatment and prognosis	Presence: 0-1; accuracy-completeness: 0-2

Clinical accuracy was judged against GeneReviews, peer-reviewed phenotypic, genetic, renal, ocular, and orthopaedic literature [8-25]. Presence and correctness were treated separately: an omitted item was scored zero rather than inaccurate, whereas an incorrect or inadequately qualified statement received one. For descriptive presentation, accuracy-completeness percentages were categorized as high (at least 85%), moderate (65.0%-84.9%), or low (below 65%); these thresholds were study-defined and not externally validated. All website scoring was performed by a single reviewer; therefore, inter-rater reliability was not assessed.

Statistical analysis

Continuous variables were summarized using means, standard deviations, and ranges; categorical results were reported as frequencies and percentages. Between-category comparisons were performed exploratorily using the Kruskal-Wallis test because the source-category groups were small and unequal. Given the very small group sizes, these analyses were considered hypothesis-generating and were not intended to establish equivalence or absence of meaningful differences between source categories; Kruskal-Wallis H statistics with three degrees of freedom are reported alongside p-values. Statistical analysis was performed using Python 3.13 (Python Software Foundation, Beaverton, Oregon) and SciPy 1.17.0 (https://scipy.org/). A two-sided p-value below 0.05 was considered statistically significant. No conventional sample-size calculation was performed because the analysis represented a census of the eligible independent pages identified in the pilot search.

Ethics and data governance

Only publicly accessible webpages were examined. The study involved no human participants, patient records, identifiable personal data, interaction with website users, or intervention. Human research ethics approval and informed consent were therefore not applicable. Local institutional governance requirements should be confirmed before submission.

## Results

Website selection and source categories

**Figure 1:**
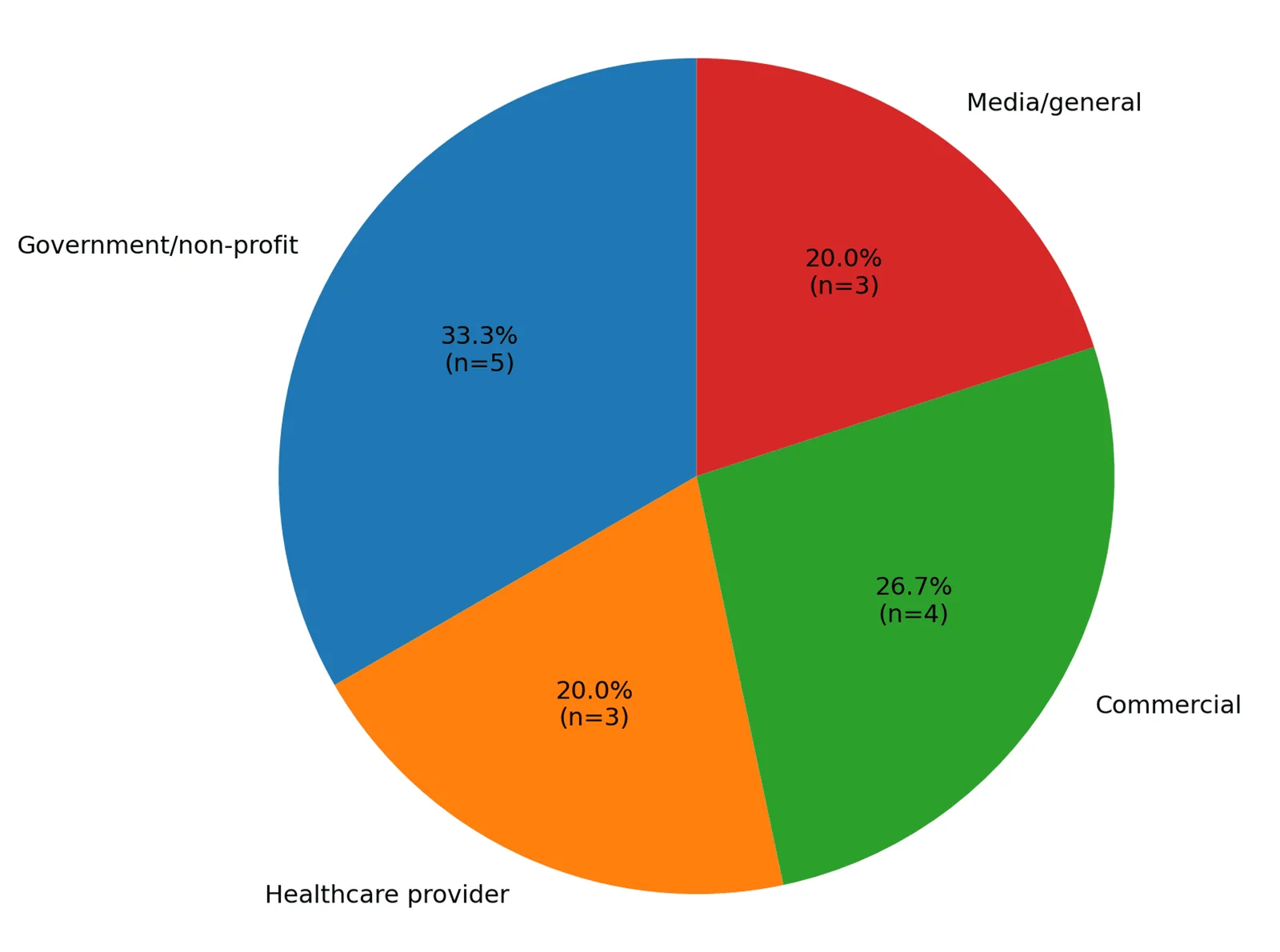
Distribution of the 15 independent patient-facing websites by source category

Twenty-four domains were screened. Fifteen independent patient-facing websites were included in the primary analysis. Of these, five (33.3%) were government or non-profit resources, three (20.0%) were healthcare-provider resources, four (26.7%) were commercial resources, and three (20.0%) were media or general-information resources (Figure 1). Three additional National Health Service (NHS)-derived or substantially duplicated pages were retained only for sensitivity analysis.

Overall and category scores

**Figure 2:**
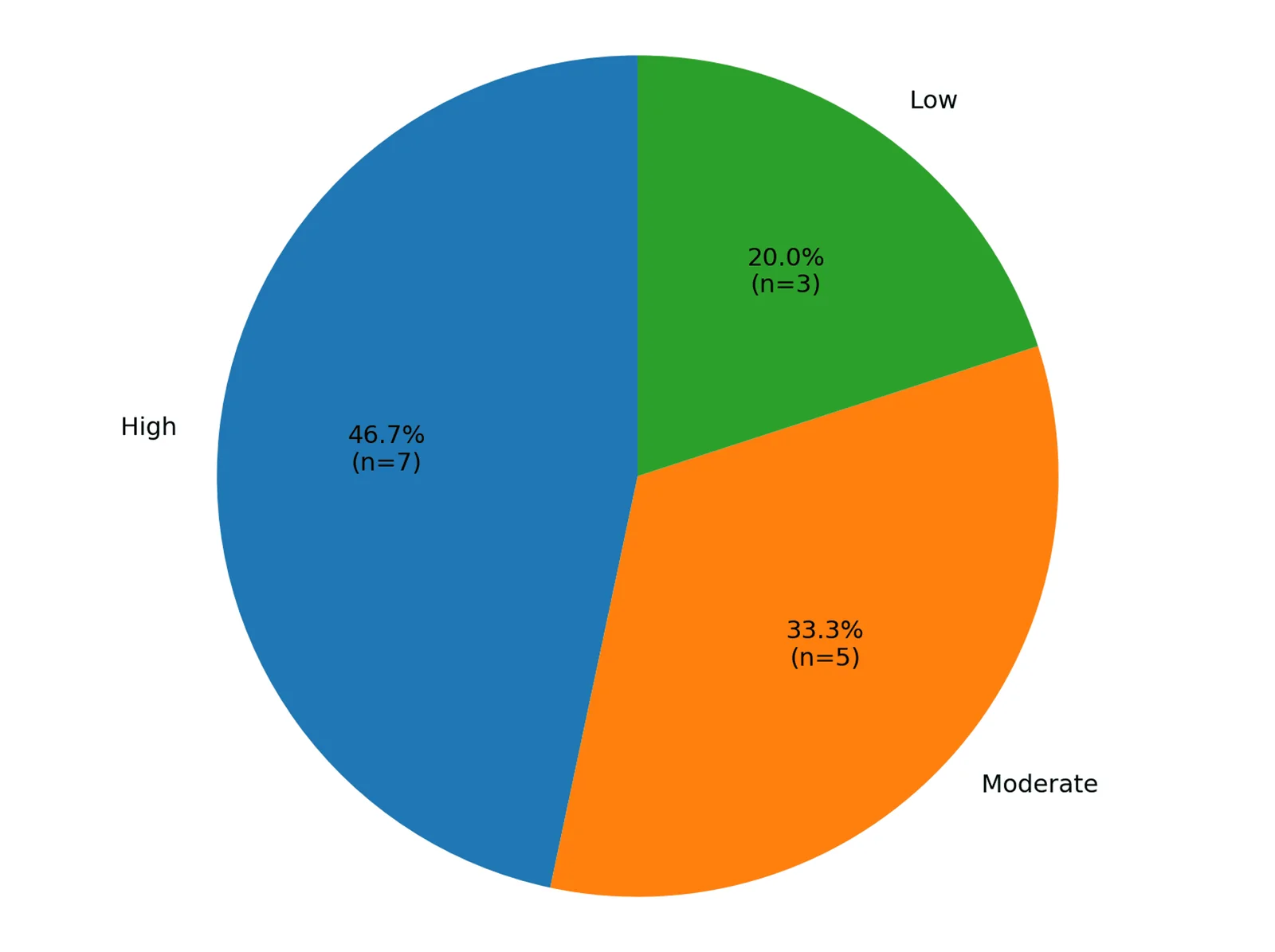
Study-defined descriptive classification of accuracy-completeness scores among the 15 primary websites

The mean presence score was 13.2 of 15 (standard deviation 1.8; range 10-15). The mean accuracy-completeness score was 23.7 of 30 (standard deviation 5.3; range 14-30), equivalent to 79.1%. Four websites covered all 15 information domains. Complete item-level scores for all 15 websites included in the primary analysis are provided in Appendices. Seven websites were classified as high quality, five as moderate quality, and three as low quality using the study-defined thresholds (Figure 2).

Descriptively, government/non-profit websites had the highest mean accuracy-completeness score (26.0 of 30), followed by healthcare-provider (25.0), media/general-information (23.7), and commercial websites (20.0). Exploratory Kruskal-Wallis tests did not detect statistically significant between-category differences in presence score (H(3)=2.25, p=0.522) or accuracy-completeness score (H(3)=2.58, p=0.462). However, the very small category sizes provided limited statistical power, and these non-significant results should not be interpreted as demonstrating equivalence or similar quality between source categories (Table 2). Given the small number of websites within each source category (n=3-5), these exploratory comparisons had limited statistical power to detect genuine between-category differences; therefore, the non-significant findings should not be interpreted as evidence of equivalent quality across source categories.

**Table 2:** Scores by website category Exploratory between-category Kruskal-Wallis tests: presence score, H(3)=2.25, p=0.522; accuracy-completeness score, H(3)=2.58, p=0.462. Because of the small category sizes, these analyses have limited statistical power and should not be interpreted as tests of equivalence.

Website category	n	Presence score, mean (SD)	Accuracy score, mean (SD)	Mean accuracy, %	Accuracy range
Overall	15	13.2 (1.8)	23.7 (5.3)	79.1	14-30
Government/non-profit	5	13.8 (1.3)	26.0 (4.1)	86.7	21-30
Healthcare provider	3	13.7 (1.5)	25.0 (4.4)	83.3	20-28
Commercial	4	12.0 (2.3)	20.0 (6.5)	66.7	14-27
Media/general information	3	13.3 (2.1)	23.7 (6.1)	78.9	17-29

Coverage and accuracy of individual topics

The classic musculoskeletal phenotype was well represented. Nail, patellar/knee, and elbow/radial-head manifestations were present on all 15 websites, and patellar/knee information was fully accurate on all pages. Iliac horns were present on 13 websites. By contrast, epidemiology was present on eight websites, and diagnosis/investigations, genetic counselling/family implications, and surveillance were each present on 11 (Table 3).

**Table 3:** Topic-level coverage and accuracy among 15 primary websites

Information domain	Present, n	Fully accurate, n	Partly accurate, n	Absent, n
Definition/multisystem nature	15	14	1	0
Etiology/LMX1B	14	11	3	1
Inheritance	14	11	3	1
Epidemiology	8	7	1	7
Age/variability	13	9	4	2
Nail manifestations	15	14	1	0
Patellar/knee manifestations	15	15	0	0
Elbow/radial-head manifestations	15	14	1	0
Iliac horns/pelvis	13	12	1	2
Renal manifestations	15	10	5	0
Ocular manifestations	14	12	2	1
Diagnosis/investigations	11	6	5	4
Genetic counselling/family	11	7	4	4
Surveillance	11	9	2	4
Treatment/prognosis	14	7	7	1

Renal manifestations were mentioned by every website, but only 10 were rated fully accurate; the remainder used incomplete or insufficiently qualified statements. Ocular manifestations were present on 14 websites, and one commercial page omitted glaucoma. Treatment or prognosis was present on 14 websites, but only seven were fully accurate. Recurrent concerns included presenting genetic testing as the sole basis of diagnosis, implying that kidney biopsy was routinely required, omitting lifelong renal or ocular surveillance, overemphasizing specialist surgery, and using unsupported or outdated numerical risk estimates.

Sensitivity analysis

Including the three substantially duplicated or NHS-derived pages increased the sample to 18 websites and raised the mean presence score from 13.2 to 13.4 and the mean accuracy-completeness percentage from 79.1% to 81.7%. This change illustrates how copied high-quality content can improve aggregate scores without representing additional independent information sources.

## Discussion

This pilot study found that online information about nail-patella syndrome was generally extensive but uneven in clinical accuracy and safety-relevant completeness. The mean presence score exceeded 13 of 15, yet the mean accuracy-completeness score was lower at 79.1%, demonstrating that mentioning a topic did not necessarily mean that it was explained correctly or adequately. The distinction between presence and accuracy is particularly important for rare diseases, where a single overgeneralized prevalence, diagnostic, or prognostic statement may shape patient expectations.

The published calcaneal-fracture study reported a mean condition-specific content score of 18.3 of 25 and found physician-authored websites to perform best [1]. In the present study, government/non-profit resources had the highest descriptive mean score, followed by healthcare-provider pages. However, each source category contained only three to five websites, substantially limiting the statistical power of the exploratory between-category comparisons and their ability to detect genuine differences. Accordingly, the non-significant Kruskal-Wallis result should not be interpreted as evidence of equivalent or similar quality across source types. Commercial resources had the lowest descriptive mean score, although performance varied within every category. Source type alone should therefore not be used as a substitute for evaluating actual content.

The strongest online content concerned the visible and radiographic phenotype. This is unsurprising because nail changes, abnormal patellae, elbow restriction, and iliac horns define the classic syndrome [8-10,21,23]. However, the clinical importance of nail-patella syndrome extends beyond orthopaedics. Renal disease may be mild and asymptomatic or progress in a subset of patients, and risk is influenced by phenotype and genotype [15-18]. Glaucoma can occur earlier than in the general population and supports continuing ophthalmological surveillance [19]. The finding that renal manifestations were universally mentioned but often incompletely explained, while glaucoma was entirely omitted by one resource, identifies a practical patient-safety gap.

Diagnostic and management information was less reliable than symptom descriptions. Nail-patella syndrome remains primarily a clinical diagnosis supported by characteristic imaging and molecular testing when appropriate [8-14]. Kidney biopsy is not required to establish the syndrome routinely, and genetic testing is not the sole route to diagnosis. Similarly, orthopaedic treatment is symptom-led and individualized; published surgical series demonstrate substantial anatomical variation and do not support a universal operation [22,23,25]. Online resources should distinguish routine surveillance from selective investigations and should avoid presenting procedures offered by a specialist centre as standard management.

This study has several limitations. First, it was a pilot search of 24 candidate domains rather than a prospectively archived, ranking-based sample drawn in fixed proportions from multiple search engines. Search results are dynamic and affected by location, personalization, and time. Search results are dynamic and affected by location, personalization, and time. Second, the primary assessment was performed once; the calcaneal-fracture model used two reviewers, and independent duplicate scoring would improve reproducibility [1]. Third, the 15-item score and the high/moderate/low thresholds were created for this study and have not been externally validated. Fourth, JAMA, DISCERN, readability, and actionability were not assessed; DISCERN was not prioritized because it focuses on treatment choices and much of the essential NPS information concerns diagnosis and surveillance [5,6]. Fifth, the sample was small, category comparisons were underpowered, and some pages were inaccessible or derivative. The 15-item NPS-specific scoring instrument was developed for this study and has not been externally validated. In addition, website scoring was performed by a single reviewer; therefore, inter-rater reliability could not be assessed. Independent duplicate scoring and external validation of the instrument would strengthen reproducibility in future studies. Finally, websites can change after assessment.

## Conclusions

This pilot study identified specific and clinically relevant gaps in online patient-facing information about nail-patella syndrome. Although the classic nail and musculoskeletal features were generally well represented, information concerning diagnosis, renal and ophthalmological surveillance, and individualized management was less consistently complete and accurate. These findings should be interpreted as identifying areas for improvement in patient-facing resources rather than establishing a definitive ranking of individual websites or source categories. Website authors should ensure that resources clearly address the multisystem nature of nail-patella syndrome, include appropriate renal and ophthalmological surveillance, distinguish routine from selective investigations, and present orthopaedic treatment as individualized rather than universal.

## Appendices

Item-level website scores

**Table 4:** Item-level accuracy-completeness scores for the 15 websites included in the primary analysis T1, definition and multisystem nature; T2, etiology and LMX1B; T3, inheritance; T4, epidemiology; T5, age and variability of presentation; T6, nail manifestations; T7, patellar and knee manifestations; T8, elbow and radial-head manifestations; T9, iliac horns and pelvic manifestations; T10, renal manifestations; T11, ocular manifestations; T12, diagnosis and investigations; T13, genetic counselling and family implications; T14, surveillance; T15, treatment and prognosis. Scoring: 0 = absent; 1 = present but incomplete, outdated, overgeneralized, or partly inaccurate; 2 = present and broadly consistent with the reference standard. The presence score represents the number of domains with a score greater than zero (range 0–15). The accuracy-completeness score represents the sum of the 15 item scores (range 0–30). These definitions match the scoring methodology in your manuscript.

Website	T1	T2	T3	T4	T5	T6	T7	T8	T9	T10	T11	T12	T13	T14	T15	Presence score (0–15)	Accuracy-completeness score (0–30)
MedlinePlus Genetics	2	2	2	2	1	2	2	2	2	2	2	1	1	0	0	13	23
GARD	2	1	2	0	2	2	2	2	2	2	2	0	1	0	1	12	21
Cleveland Clinic	2	2	2	0	2	2	2	2	2	2	2	1	2	2	2	14	27
NORD	2	2	2	2	2	2	2	2	2	2	2	2	2	2	2	15	30
Contact	2	2	2	0	2	2	2	2	2	1	2	2	2	1	2	14	26
Orphanet	2	2	2	2	2	2	2	2	2	2	2	2	2	2	2	15	30
Osmosis	2	2	2	0	1	2	2	2	2	1	2	1	2	2	1	14	24
Medical News Today	2	2	2	1	2	2	2	2	2	2	2	2	2	2	2	15	29
News-Medical	2	0	1	0	1	1	2	2	0	2	2	0	1	2	1	11	17
Wikipedia	2	1	2	2	2	2	2	2	2	1	2	1	0	2	2	14	25
Paley Orthopedic & Spine Institute	2	2	2	2	1	2	2	2	2	1	1	0	0	0	1	12	20
International Center for Limb Lengthening	2	2	2	2	2	2	2	2	2	2	2	2	1	2	1	15	28
MSD Manual Consumer Version	2	1	0	0	0	2	2	2	1	2	1	1	0	0	1	10	15
Syndrome.co.uk	2	2	1	0	2	2	2	2	2	2	2	2	2	2	2	14	27
Healthera	1	2	1	2	0	2	2	1	0	1	0	0	0	1	1	10	14
